# Introducing all-inkjet-printed microneedles for in-vivo biosensing

**DOI:** 10.1038/s41598-024-80840-1

**Published:** 2024-12-02

**Authors:** Giulio Rosati, Patricia Batista Deroco, Matheus Guitti Bonando, Gustavo G. Dalkiranis, Kumara Cordero-Edwards, Gabriel Maroli, Lauro Tatsuo Kubota, Osvaldo N. Oliveira, Lúcia Akemi Miyazato Saito, Cecilia de Carvalho Castro Silva, Arben Merkoçi

**Affiliations:** 1 Catalan Institute of Nanoscience and Nanotechnology (ICN2), CSIC and BIST, Campus UAB, 08193 Bellaterra, Barcelona Spain; 2 Institute of Chemistry, University of Campinas – UNICAMP, Campinas, 13083-970 Brazil; 3 National Institute of Science and Technology in Bioanalytic (INCTBio), Campinas, 13083-970 Brazil; 4 School of Engineering, Mackenzie Presbyterian University, Rua da Consolação, 930, Sao Paulo, SP 01302-907 Brazil; 5 MackGraphe – Mackenzie Institute for Research in Graphene and Nanotechnologies, Rua da Consolação, 930, Sao Paulo, SP 01302-907 Brazil; 6 São Carlos Institute of Physics, University of São Paulo, P.O. Box 369, São Carlos, SP 13560-970 Brazil; 7 Instituto de Investigaciones en Ingeniería Eléctrica Alfredo Desages (IIIE), Universidad Nacional del Sur, CONICET, Avenida Colón 80, Bahía Blanca, Buenos Aires Argentina; 8 ICREA, Passeig Lluís Companys 23, 08010 Barcelona, Spain

**Keywords:** Microneedles, Inkjet printing, Silver nanoparticles, Plants, Precision agriculture, EIS, Surface patterning, Sensors and biosensors, Biomedical engineering

## Abstract

Microneedles are mainly used for pain-free drug administration and in biosensing for wearable systems. They are also promising for fields such as agronomy for precision farming, but their fabrication is not straightforward, often requiring expensive equipment and cleanroom protocols, being unsuitable for mass production. Here, we report a new and simple method for the scalable fabrication of all-inkjet-printed conductive microneedles based on silver nanoparticles (extensible to any other metallic nanoparticle ink) and a simple example of their application for monitoring the electrochemical properties of plants.

## Introduction

Microneedles proved helpful for minimally invasive sampling^1,2^, diagnostics^3,4^, biosensing^5–8^, monitoring^9–12^, and drug delivery^13^. They hold great potential not only in medicine but also in agronomy for precision farming^14,15^. They are still not pervasive because of their cumbersome and difficult-to-scale-up fabrication. In principle, mass production could be viable with inkjet printing already employed in flexible electronics and biosensing owing to its features, such as non-contact mask-less deposition of nanofunctional inks and ultrafast concept-to-prototype time. In fact, microstructures in the low-micrometer range have been produced with 3D inkjet metal nanoparticles. However, this has been possible only with very advanced equipment^16–19^.

Microneedles were first made from silicon as the microelectronics industry provided tools for manufacturing integrated circuits that could be adapted to microneedle fabrication and silicon is still the most common microneedle material^20^. However, cleanroom-based fabrication requires complex operations and high costs to achieve mass production. Furthermore, silicon has several disadvantages for wearable applications, which is why polymer materials, metals, and other materials such as ceramics have been investigated for microneedles fabrication. For the polymers-based microneedles, it is increasingly clear that the favoured fabrication methods used to develop the next generation of polymer microneedle point-of-care tests and drug delivery patches will be photolithography, replica moulding, 3D printing, and micromachining^20^. For the metal microneedles, photochemical etching, electroplating, and laser cutting are the most common fabrication techniques^20^. Unfortunately, from the manufacturing perspective, the fabrication of metal microneedles has complexities like electroplating and lift-off, which are undesirable for mass production^20^. Other manufacturing processes for microneedle fabrication include injection moulding, wet chemical etching, reactive ion etching, hot embossing, laser drilling, lithography plus electroforming, drawing lithography, two-photon polymerization, and 3D printing^20^.

In recent years, additive manufacturing (3D printing) has gained attention as a means of producing MN arrays. A 3D printer builds an object by depositing the desired material layer-by-layer^21^. Generally, three types of 3D printing have been studied, including stereolithography (SLA), fused deposition modelling (FDM), and digital light processing (DLP). The accuracy of FDM printing technology is affected by several factors, such as temperature and the size of the release nozzle, and the minimum feature size for these two methods can be 100 μm. The DLP system has a high printing accuracy and can meet the manufacturing accuracy of the microneedle^22^. Recent commercialization of the 2PP microtechnology by companies such as Nanoscribe GmbH of Karlsruhe, Germany, with their Photonic Professional GT system, has enabled the precise manufacture of devices at submicrometer resolution. This technology enables reproducible production of complex structures in a short manufacturing time and with exceptional flexibility, using femtosecond laser pulses from a near-infrared (NIR) laser beam via a controlled printing head to selectively polymerize an uncured photosensitive resin^23^. Unlike FDM, material jetting (MJ) consists of thermal or piezoelectric printheads where a liquid building material is deposited dropwise at a high speed. The predominantly used ink for MJ, ultraviolet (UV) curable ink, then requires a short exposure to UV light for curing before deposition for the next layer can proceed. This process is repeated until the whole model is built^24^.

All these additive manufacturing methods rely on polymer materials, which need to go through a second step to be plated or covered with a conductive material to be used for biosensing purposes. The only single-step method for the fabrication of metal microneedles available to the knowledge of the authors is micromilling of metal substrates (e.g. stainless steel). However, this method would wear or even break the tool used for the milling. Also, the production time is too long, making it economically unsuitable, and it is difficult to convert multi-step batch processing to high-throughput manufacturing scalability^22^. Moreover, this method does not allow the fabrication on flexible substrates since it is performed on a solid block of metal.

In this work, we propose a new approach for the single-step fabrication of microneedles using 3D inkjet-printed silver nanoparticles with commercial ink and a widespread inkjet printer as a proof-of-concept of the feasibility of this approach for the scalable fabrication of devices with applications in healthcare and agronomy.

**Fig. 1:**
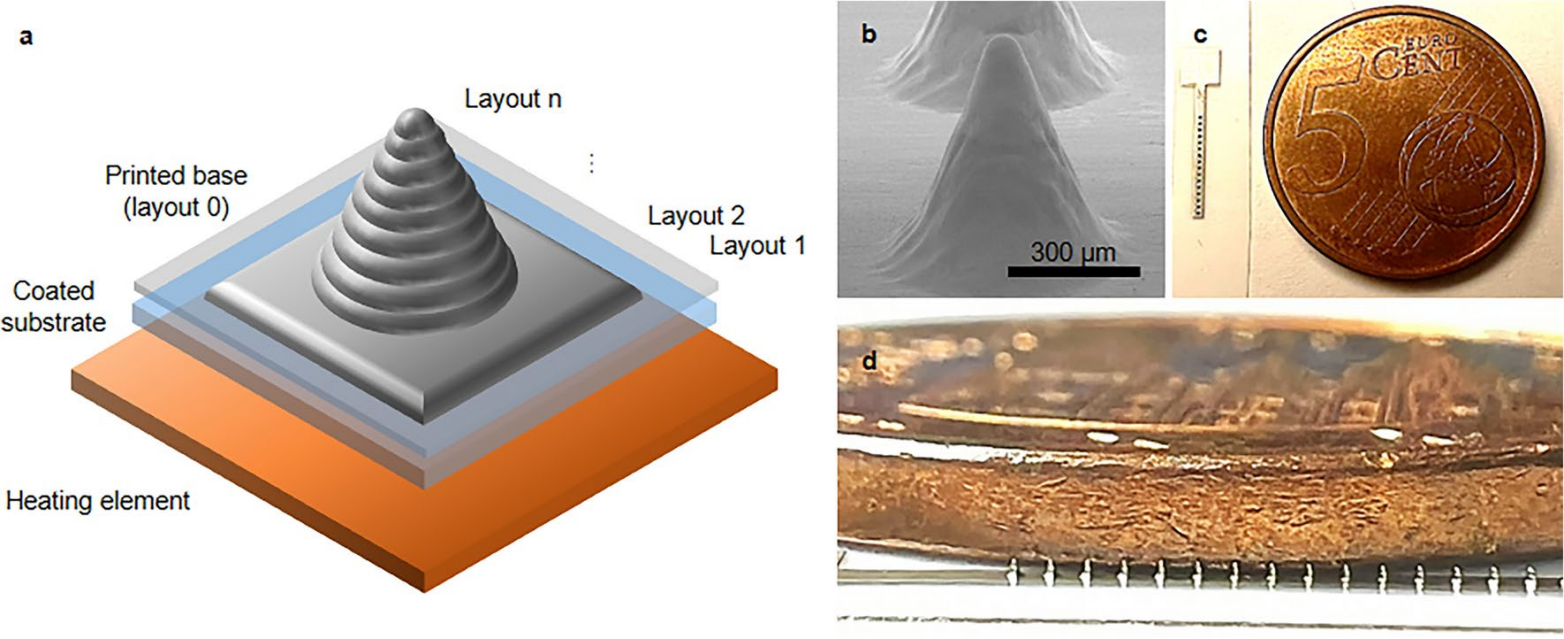
Inkjet-printed fabrication strategy for microneedles. (**a**) Schematic of the final microneedle 3D layout obtained with the inkjet 3D fabrication method. (**b**) SEM picture of a real inkjet-printed 3D microneedle. (**c**, **d**) Dimensional comparison of a 100 µm high microneedles array of 16 inkjet-printed microneedles next to a 5 euro cent coin.

Inkjet printing of metal nanoparticles-based inks is used routinely to fabricate thin-film flexible electronics and biosensors on plastics and other substrates. Standard inkjet printing methods are based on the drop-on-demand method, so picoliter drops are jetted from the printhead onto the substrate, defined by the layout. If the substrate is absorbent (e.g., paper or mesoporous-coated plastics), the droplets spread on its surface and dries faster, thanks to the higher surface-to-volume ratio of the substrate structure. If the substrate is impermeable (e.g., normal plastics), the droplets accumulate in one or multiple drops depending on the surface energy of the substrate and eventual priming procedures performed on it. For nanofunctional inks based on metal nanoparticles, the thickness of the deposited metal nanoparticles layer depends on the droplets’ volume, the type of substrate, the nanoparticle content of the ink, and the number of printed layers. However, there is a lower limit in the x–y dimension of the printable structures due to the formation of large drops when the jetted droplets accumulate onto the substrate. There is also an upper limit for the height that can be reached due to the x–y dispersion of the ink in liquid form on the substrate when multiple layers are printed. That is why these structures cannot be defined as 3D or vertical, and are typically called 2.5D. A useful example comes from FDM 3D printing. With this widespread method, polymer filaments are taken close to their melting temperature and extruded through a nozzle over a plate following a 2D design. Notably, the heat is applied to the filament right before pushing it out of its nozzle. The polymer solidifies very rapidly out of the nozzle onto the substrate, and the process is repeated for multiple layers with different designs. Each 2D design is obtained by segmenting a 3D one into layers as thick as the extruded filament to obtain the 3D object, with each layer being printed and sticking over the previous one. To inkjet-print a 3D metal nanoparticles object, thus having vertical dimensions comparable with the horizontal ones, we have taken the core characteristic of standard 3D printing into a research-grade inkjet printer. We have added a thin heater element under our printing substrate to immediately cure the ink picoliter droplets once reaching the substrate, and we have programmed the inkjet printer to print multiple 2D designs and create vertical structures of different shapes (Fig. 1).

In our approach, the inkjet-printed nanoparticles ink is jetted onto a substrate heated to a temperature sufficient to rapidly cure the jetted drops before they can drip down. In this way, the solvent of the ink evaporates as soon as the drop enter in contact with the substrate or the previously printed structure, leaving the nanoparticles exactly where the drop has been jetted. Therefore, differently from FM 3D printing, in our case the heat is applied to the substrate, and not to obtain the filament melting, with the ink droplets rapid curing. This is a crucial difference since our starting material is not solid but liquid (the ink). Additionally, with our method the created structures are immediately conductive being metallic (different metals are possible depending on the nanoparticles chosen for the ink). All the other 3D printing methods, such as SLA, 2PP, and DLP relies on the solidification of liquid polymers in specific areas layer-by-layer, which is a rather similar concept to our approach. However, for all of them the process is mediated by light producing a polymer that needs further treatments or plating to be made conductive. As in standard 3D printing, temperature control is crucial. In general, if the temperature is too low, the droplets will remain liquid, limiting or completely hindering the structure’s verticality. In contrast, the ink would dry inside the printhead nozzle at too high temperatures, clogging them definitely. Furthermore, we tried to avoid high temperatures to keep the method open for sensitive substrates such as plastic and paper.

## Results

### Geometrical characteristics

**Fig. 2:**
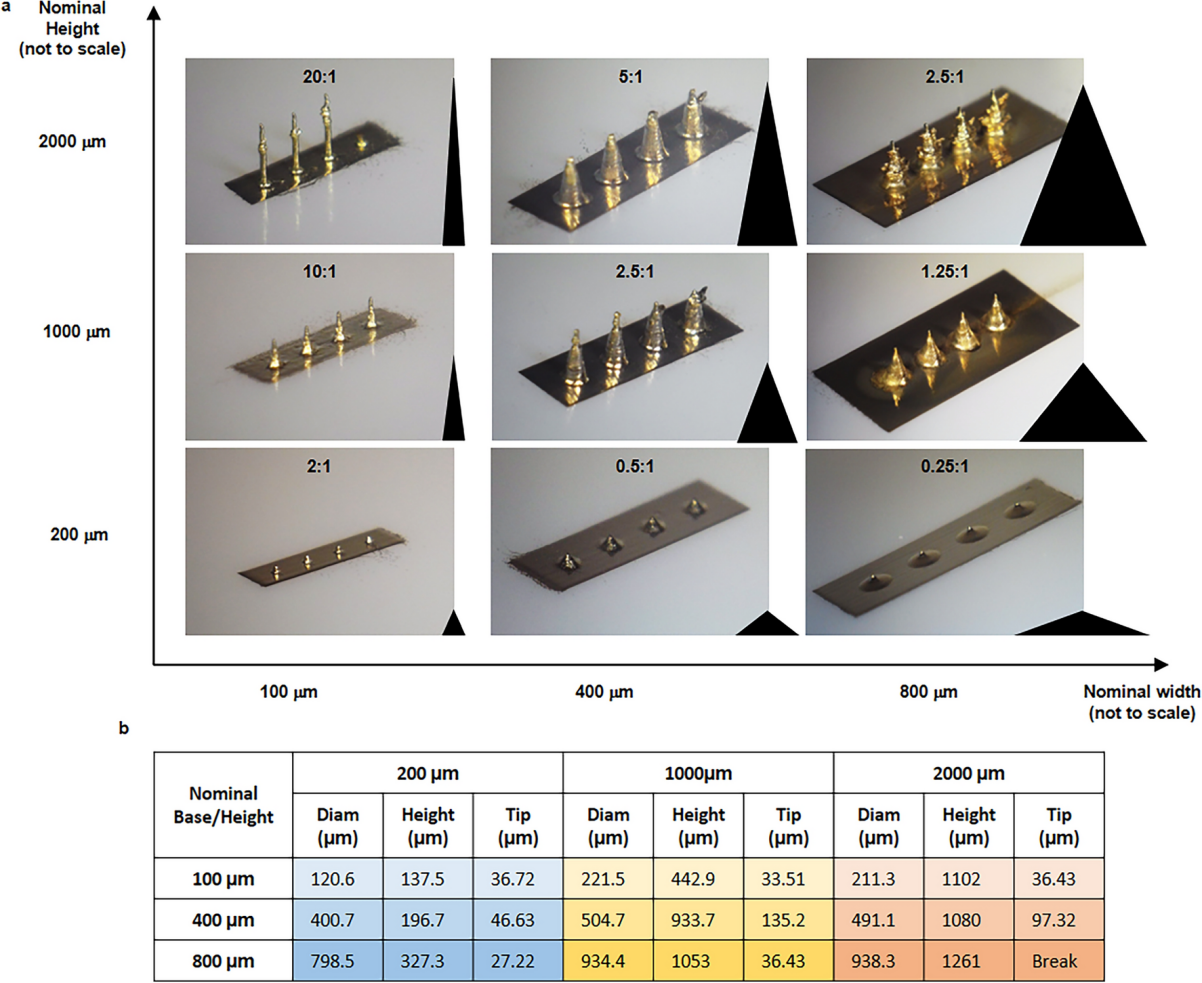
Geometric study of the printable height/width ratio for the microneedles. (**a**) Circular-based microneedles have been printed with height/width ratio between 0.25 and 20 with base widths ranging from 100 to 800 µm. (**b**) Summary of the measured base, height, and tip width.

The achievable geometrical characteristics of the 3D printable structures resulted very interesting with our layer-by-layer inkjet method. We started characterising circular-based microneedles focusing on their base width (diameter) and height. As depicted in Fig. 2, almost vertical structures with a height/width ratio of 20 can be fabricated even with a base diameter of 100 µm (Fig. S1). However, as indicated in the table in Fig. 2b, the adherence to the nominal dimensions is inversely proportional to the needles’ height. This happens because the further we print from the surface; the slower the curing is, being far away from the heater under the substrate. Furthermore, the quality of the needles depends on the stability of the ink and its printing during the whole process. Satellite drops formation or misalignment of the drops out of the nozzles are detrimental.

### Mechanical and electrical characterization

**Fig. 3:**
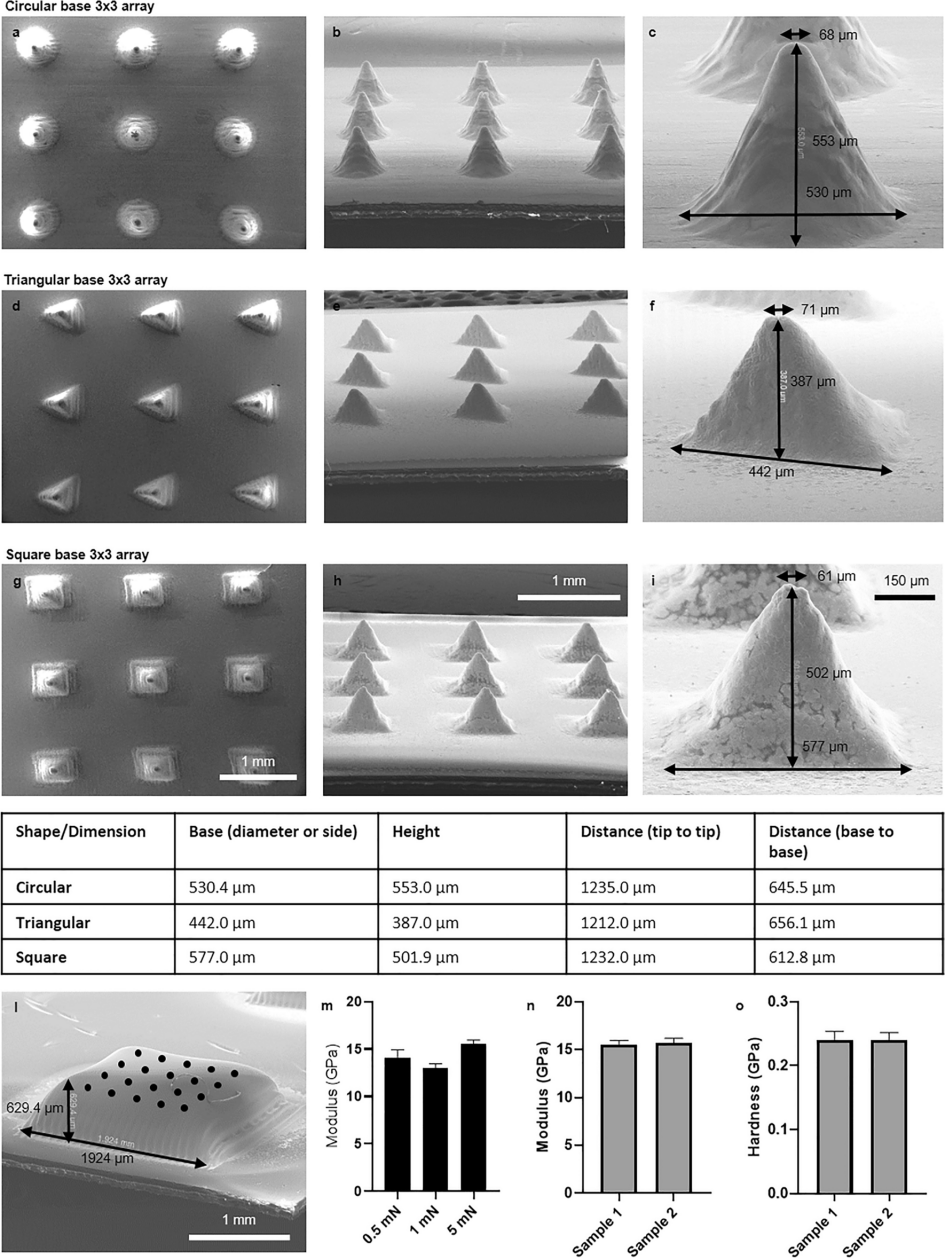
Geometric study of the microneedles’ printable base shapes and their mechanical characteristics. (**a**, **d**, **g**) SEM pictures of 3 × 3 microneedles arrays seen from above. (**b**, **e**, **h**) SEM pictures of the 3 × 3 microneedles array seen from the side with a 30 degrees tilt. (**c**, **f**, **i**) SEM micrographs of a single microneedle of the array with its base width, height, and tip width measurements. All the geometrical characteristics of the 3 × 3 microneedles array are summarized in the table (all the measurements are in µm). (**l**) SEM picture of one of the samples tested to measure the material’s hardness and Young modulus with the 5 × 4 testing grid. (**m**) Average and standard deviations of the Young modulus obtained from the nanoindentation measurements with forces ranging from 0.5 to 5 mN. (**n**, **o**) Comparison of the Young modulus and the hardness average values obtained on two samples.

The following step has been to investigate the possible shapes of the inkjet-printed microneedles (Fig. 3a–i) and their mechanical characteristics. The shapes and dimensions of the 3 × 3 arrays were reproducible and in line with the ones typically used for biomedical applications. The minimum width of the microneedle tips ranged from 60 to 70 µm for any selected shape. The determination of the mechanical properties of the microneedles is generally complicated because they depend on both the material and the layout. Therefore, we tested the mechanical characteristics of the material alone using rectangular-based 3D inkjet-printed solids following a 5 × 4 (N = 20) pints grid (Fig. 3l). They were tested by nanoindentation with a Berkovich diamond tip applying forces ranging from 0.5 to 5 mN (Fig. 3m).

The average Young modulus of our 3D inkjet printed AgNP-based material was 15.6 GPa (Fig. 3m,n), i.e., almost 20% of the value for bulk silver, even without any thermal or photonic post-treatment after printing. The used forces did not evidence relevant variation in the obtained values. The average hardness resulted 0.24 (Fig. 3o), 76% the one of bulk silver. Different samples gave very similar results (Fig. 3n,o).

Regarding the electrical characterization of the MNs, the resistance of a 600 µm high microneedle with a base of the same diameter and a 30 µm tip has been measured manually placing the probes from the printed platform close to its base to the tip. The resistance was 110 mΩ. The resistance between opposite corners of the square printed platform at the base of the MNs was 86 mΩ. Considering the low values obtained, no further characterizations have been performed since these values are perfectly in line with the intended electrochemical applications.

### Application case study: plants impedimetric measurements

**Fig. 4:**
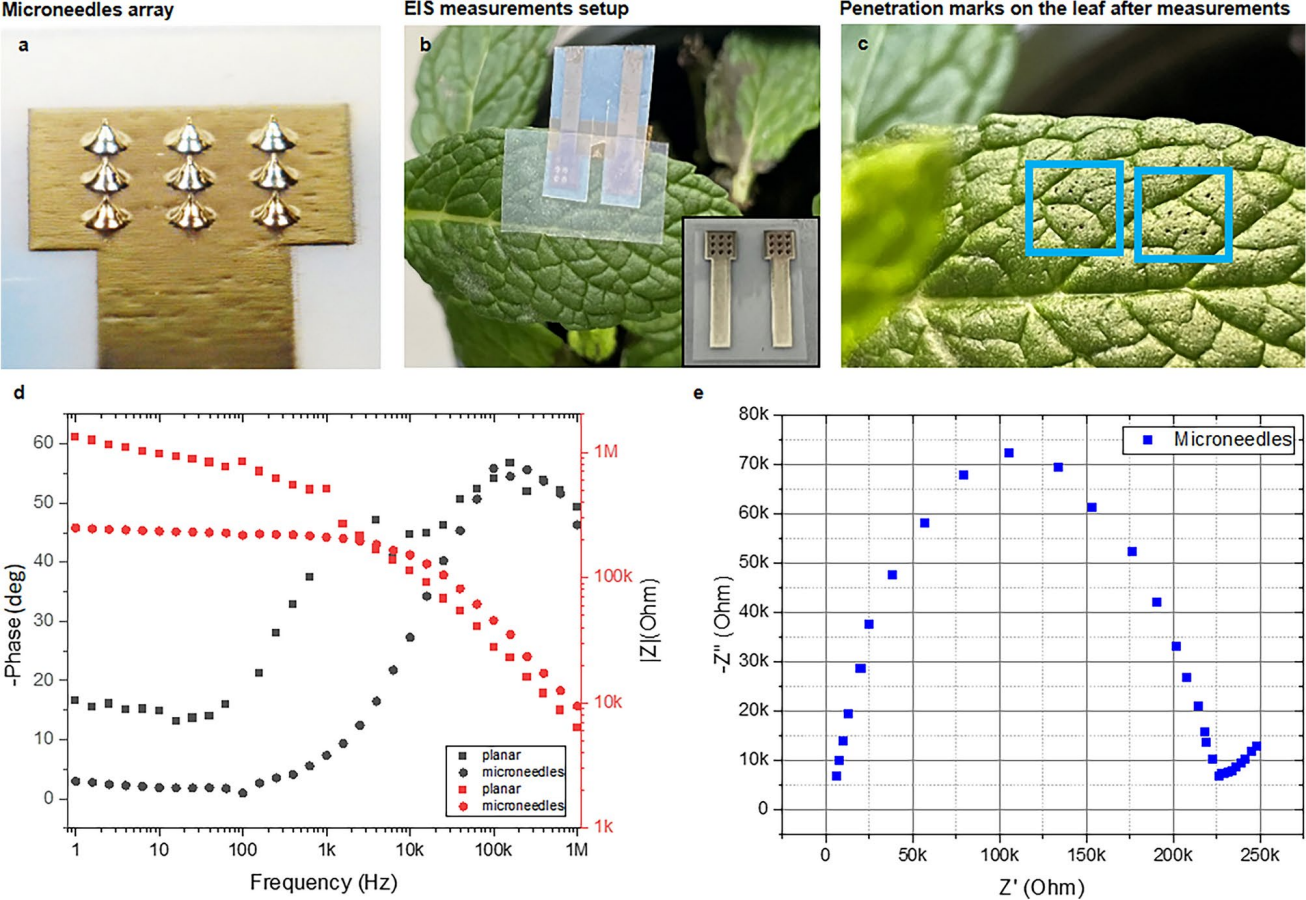
Electrochemical impedance spectroscopy response of two adjacent 3 × 3 microneedles arrays in a mint leaf. (**a**, **b**, **c**) Pictures of the 100 µm high microneedles 3 × 3 array, its use for EIS measurements onto a mint leaf, and the penetration marks left on the leaf after the measurements. (**d**) Bode diagrams of the microneedles and planar electrodes EIS responses compared (the most noisy values for the planar electrode phase have been removed). (**e**) Nyquist plot of the microneedles response.

Finally, to test the microneedles applicability for in-vivo measurements, two 3 × 3 arrays of 100 µm high microneedles have been fabricated on top of an AgNPs square base (Fig. 4a) and their resilience to the penetration of leaves have been successful. We have observed clear marks only on porcine skin and mint leaves (Fig. S2). No evident damage to the 3 × 3 microneedles array was observed. Appropriate tuning of the height of the microneedles may give access to any compartment of the leaf’s internal structure. Two microneedles arrays have then been placed next to each other on top of a mint leaf (Fig. 4b,c). Impedance spectroscopy measurements were compared with those obtained with the same silver rectangular base but without microneedles, performed as control tests. As expected, the impedance recorded with the microneedles is much lower (Fig. 4a) due to the penetration of the cuticle (the most external leaf layer), which typically acts like an insulator and presents a typical Randle’s cell response (Fig. 4d) in the measurements performed with the planar electrodes. The MNs showed a similar response but almost one order of magnitude lower at low frequencies. Furthermore, the recorded EIS response with the MNs has been less noisy and better defined at the lowest frequencies, showing an almost perfectly resistive behaviour under 1 kHz. This allows direct access to the plant’s internal compartments and structures, paving the way to the real-time recording of their electrical and electrochemical characteristics by using needles of different heights, i.e., different penetration depths. Further refinement of the fabrication and inclusion of other metal nanoparticles, such as gold, may also give the opportunity to functionalize the microneedles tips for detecting specific biomarkers and monitoring over time.

## Discussion

### Comparison with other MNs fabrication methods

The results clearly demonstrate that this single step fabrication method we propose allows controlling the geometric characteristics (dimensions and shapes) of AgNP conductive microneedles in compliance with the requirements of most of the biomedical applications of these architectures. Furthermore, the microneedles reported in this work are mechanically stable and immediately conductive after fabrication without any need for post-printing treatments, such as curing or sintering. The equipment used for the fabrication is widespread in most laboratories dedicated to printed electronics and has been modified solely by integrating a commercially available heater on the printing plate. Therefore, we envision that the method we propose (protocols reported in the supplementary information) will allow a significant advancement in the fabrication of these important architectures. Layouts modifications will also be easily implementable simply changing the layers structures accordingly. No a priori limitations on the type of materials for the microneedles are highlighted, with the only important condition related to the stable printing of the material over the whole printing period (few hours). Table 1 brings a summary of the characteristics of our fabrication method compared with the most widely used ones. The comparison has been driven on the base of the minimum MNs tip dimension achievable, the time per MN (considering if the method allows for the fabrication of the MNs in parallel, i.e. all at the same time, or if it is performed one MN per time). Then, it has been considered if the MNs that can be produced with each fabrication method can be both hollow and solid or only solid and if they are conductive (without further treatments). As made evident from this comparison, the inkjet fabrication method we propose is the only single-step technique allowing for the fabrication of conductive MNs on flexible substrates with a relatively low time per MN. In fact, it should also be considered that using multinozzle printheads the parallel fabrication of a reduced number of MNs is possible (the time per MN is discussed more in depth in the next paragraph). Furthermore, the resolution obtained is in line with the requirements for biomedical and agronomy applications^25^ (Fig. 4c, S2) and in general lower than most of other additive manufacturing methods.

**Table 1:** Comparison of the characteristics of the main fabrication methods for MNs.

Category	Fabrication method	Material	Tip min. dimension (µm)	Min. steps required	Time/MN	MNs type	Conductive MNs	References
Lithography	Wet chemical etching	Silicon	< 1	> 2	min (P)	S/H	Semicond	^20–22^
	Reactive ion etching		< 1	> 2	min (P)	S/H	Semicond	^20–22^
	Lithography + electroforming		< 1	> 3	min (P)	S/H	Semicond	^20–22^
	Drawing lithography		< 10	> 2	min (P)	S/H	Semicond	^20–22^
	Photochemical etching	Metal, Silicon	< 1	> 2	min (P)	S/H	Semicond	^20–22^
Moulding and drilling	Hot embossing	Polymers	< 1	1	sec (P)	S/H	No	^22^
	Injection molding		< 1	1	sec (P)	S/H	No	^22^
	Micromilling	Polymers and metals	≈ 30	1/2	min (OPT)	S (H)	Yes, solid substrate	^22^
	Laser drilling		≈ 30	2	sec (OPT)	S	Yes	^22^
3D printing	SLA	Polymers	80	1	min (OPT)	S/H	No	^22,23^
	FDM		170–220	1	min (OPT)	S	No	^22,23^
	DLP		40	1	min (OPT)	S/H	No	^22,23^
	2PP		6	1	min(OPT)	S/H	No	^22–24^
	Printing of AgNPs	Metals	30	1	min (SPT)	S	Yes, thin substrate	This work

*P* fabricated in parallel, *OPT* fabricated one per time, *SPT* fabricated some per time, *S* Solid, *H* Hollow microneedles.

### Curing of the jetted ink at different heights

Differently from FDM 3D printing, where the heat is applied to the polymer by the extruder which is always standing at the top layer of the 3D printed structure, in our method the heat is applied at the base of the structure. Consequently, at different heights the actual temperature of the structure may vary significantly. Since the temperature of the structure at the ink droplets point of impact is responsible for the real-time curing of the ink granting the good resolution of the process, this variation could be detrimental if not controlled. In our work, this effect has been reduced by slightly increasing the temperature of the heater from 88 °C up to 95 °C proportionally with the height of the printed structures. Furthermore, it has been proven that the abovementioned lack of reproducibility is found only in very vertical structures, i.e. structures with small base areas (Fig. 2a,b). This is reasonable, considering that the wider the base the higher the thermal conductivity of the printed MNs silver structure, thus the higher the temperature at the tip. Experimentally, an approximate ratio of 2 between the height and the base diameter of the MNs has been verified to yield reliable structures dimensions. However, this is true only for structures with a base width over 100 µm (due to the minimum resolution of the printer) and a height below 1000 µm (due to the decrease of the temperature with the distance from the substrate).

### Scalability

One of the characteristics of utmost importance for microneedles to reach the market is the scalability of the process and its costs. We have analysed these aspects for our 3D inkjet printing method and compared them with the most typical currently used approaches. Some considerations are needed: i. the method we propose is direct, waste-free, and can take place out-of-the-cleanroom; ii. the cost of the equipment required is relatively low (a research-grade X–Y printer typically costs in the range between 15,000 and 35,000 Euros); iii. The number of microneedles which can be fabricated per batch depends uniquely on the dimension of the printing bench (A3 for the Dimatix printer) and on the dimension (or number) of the heating modules (and of the respective power supplies); iv. The required number of layers is high, but the fabrication procedure can be automatized, and the use of multiple nozzles can drastically reduce the time needed for the printing of each layer; v. The fabricated microneedles will be thermally and electrically conductive without the need for any additional treatment or process, opening up for a variety of applications.

In this work, we used a 50 × 50 mm heating module and only 1 of the 16 nozzles in the Dimatix inkjet printer. The average fabrication time for each microneedle was about 30 min, but using all the printhead nozzles, this time would be lower than 112 s. The process may be scaled up using the whole A3 area available for printing in the Dimatix printer (with a suitable number of heaters or using a custom-sized heater covering the entire area). Furthermore, other printers on the market with a greater number of nozzles and a higher speed in the X–Y movement could probably go below 15 s per microneedle. To our knowledge, this method is the fastest and most direct method for the scalable single step fabrication of conductive solid microneedles in the literature.

### AgNPs structures oxidation potential

As it is well known, silver is a metal prone to oxidation even at room temperature and ambient pressure with time, producing a dark thin layer, limiting its conductivity. The process is favoured by temperature. However, this happens only for temperatures over 150 °C and it finds its peak around 300 °C^26^ even in presence of atomic oxygen. During the MNs inkjet fabrication we used a temperature of 88 °C and we never observed neither in the microscopic characterization nor during the electrical or electrochemical characterizations any relevant oxidation of the MNs surface.

### Plants EIS testing and potential applications

Despite being only a case study application to prove the validity and the good electrochemical properties of our MNs, the EIS testing on plat leaves marked a significant milestone for our devices. The incorporation of MNs in agriculture is increasingly acquiring importance both for sensing and biosensing, and for efficient drug delivery reducing soil pollution^27–29^. With the control our method allows for the MNs structure and geometrical characteristics, customizing the MNs dimension to target specific compartments of leaves of plants of different species is a realistic scenario. This paves the way to an efficient control and monitoring of the plants status. Ion-selective wearable microneedles have been fabricated by other groups for human use^30^. The same concept but with our fabrication may be used for plants to monitor their nutrients uptake and minimize the use of fertilizers (and soil stress) to what is really needed.

Finally, the implementation of wireless and battery-free smartphone interface systems, as recently done in our group for wearable devices in biomedicine^31^ would allow an easy and eventually disposable use of devices fabricated with our technology.

## Materials and methods

### Design of the microneedles layout

The layouts have been designed in Corel Draw® software and exported in BMP files; then they were uploaded to the Dimatix printer software. Each layout was printed at least ten times to obtain a significant increase in the height of the microneedles. A different number of printing layers could be performed to obtain different 3D profiles.

### Printing protocol

The inkjet printer used in this study is a Dimatix 2830 (FUJIFILM Dimatix Inc., 2006), with the substrate plate always set at the maximum temperature (60 °C), modified simply by adding a heater on the top left corner of the plate and keeping it in position with Kapton tape. The heater under the substrate was a commercial 50 × 50 mm flexible silicone heating element RS Pro 245–506 (12 V DC, 2.5W), and the maximum temperature achievable without the clogging of the printhead or deformations of the substrate was around 88 °C. This was obtained by supplying the heater with a voltage of 16 V and a DC current of 0.28 A (approximately 4.5 W) from an Agilent triple output DC power supply model U8032A (0-60V, 3A/5V). The printer has been placed on a marble table to reduce the effect of eventual floor vibrations. The power of the heater was slightly changed with the increase of the printed number of layers to cure the ink fast, even when deposited at a greater distance from the base of the microneedle. Tables indicating the protocols to obtain the circular-based microneedles can be found in the supplementary materials (Tab. S1-S9).

The most advantageous substrate and ink have been Mitsubishi Paper Mills coated transparent PET substrate (NB-TP-3GU100) and Novacentrix JS-A102A AgNPs ink. This was owing to the mesoporous alumina coating on the substrate, which drains the ink solvent of the first (and bigger) layers, thus significantly reducing their drying time. Furthermore, the Mitsubishi substrate coating contains a chemical agent that is supposed to help remove the organic coating from the silver nanoparticles to increase the printed structure conductivity in a chemical sintering process. Unfortunately, Mitsubishi has not disclosed the chemical agent. The Novacentrix ink has been selected because it is easily printable and stable. This last characteristic is fundamental since the required number of layers to print for a 1 mm high microneedle with a 100 µm base is 750. Nevertheless, any other stable metal nanoparticle ink may be used to fabricate the inkjet-printed microneedles since no a priori factors prevent it. Before the printing starts, the Novacentrix ink has been filtered with a hydrophilic PTFE membrane syringe 0.2 µm filter. The droplets jetted by the best-performing printer’s nozzles on each occasion have been aligned.

### Optical, mechanical and electrical characterizations

The SEM analysis was performed by an ESEM Quanta 650 FEG microscope. The images were collected operating in a high vacuum with an electron beam energy of 20 kV and secondary electrons in-lens detector. All the microneedles dimensions were measured with the microscope software.

The nanoindentation technique tested the mechanical properties using an iNano from the KLA instrument equipped with a Berkovich pyramidal-shaped diamond tip. Experiments were carried out in the static (ISO 14,577) mode, and the thermal drift was always kept below ± 0.05 nm s^−1^ . Three different loads (0.5, 1, and 5  mN) were applied. To ensure statistical robustness and accuracy of the results, a total of 20 indents per load were performed in four samples. Indents were spaced 20 μm apart, providing sufficient independence in all cases.

The electrical resistance measurements have been performed with a Keithley DMM6500 6 1/2 digit multimeter, positioning the probes on the base and the tip of a 600 µm high MN with a base width of 600 µm and a tip diameter of 30 µm.

### Impedance measurements on plants

3 × 3 arrays of 100 µm high circular-based microneedles (100 µm base diameter and 30 µm tip diameter) were fabricated on top of an AgNPs square base (Fig. 2b inset). Two of them have been placed next to each other at a distance of 2 mm on top of a mint leaf. No passivation of the silver surface was performed, considering that the leaf cuticle (the leaf most external layer), if not penetrated, acts like an insulator due to its wax content. Control measurements have been performed using identical AgNPs square bases at the same distance, but without microneedles printed on top, thus in direct contact with the leaf but without any penetration. Impedance spectroscopy measurements were performed using a PalmSense 4 potentiostat connecting the microneedles and the planar patches with crocodile clamps. The measurements were performed in the 1 Hz to 1MHz frequency range (5 points/decade) with a 0 V DC bias and 10 mV AC.

## Supplementary Information

Supplementary Information.

## Data Availability

The main data supporting the results of this study are available within the paper and its Supplementary Information. Layouts for printing the microneedles following the layers tables in the supplementary information will be available upon request.
