# Supplementary material for: Introducing all-inkjet-printed microneedles for in-vivo biosensing
Source: Sci Rep. 2024 Dec 2;14:29975. doi: 10.1038/s41598-024-80840-1 (PMC11612178; doi:10.1038/s41598-024-80840-1)
Supplement: Supplementary file 1 — Supplementary Information. [file 41598_2024_80840_MOESM1_ESM.docx]

**Supplementary information**

**Introducing all-inkjet-printed microneedles for in-vivo biosensing**

Giulio Rosati^a^*+, Patricia Batista Deroco^a,b,c^+, Matheus Guitti Bonando^a,d,e^+, Gustavo G. Dalkiranis^a,f^, Kumara Cordero-Edwards^a^, Gabriel Maroli^a,g^, Lauro Tatsuo Kubota^b,c^, Osvaldo N. Oliveira Jr.^f^, Lúcia Akemi Miyazato Saito^d,e^, Cecilia de Carvalho Castro Silva ^d,e^, Arben Merkoçi^a,h^*

**Protocols: layers tables for circular MNs**

In the following we summarize the protocols followed for the fabrication of the tested MNs. A different layout has been prepared for each step, consisting in an array of black figures (i.e. circles, squares, triangles) with different dimensions, as indicated in the base diameter column. Each of these layouts have been printed for a different number of times (# of layers), setting the substrate thickness parameter of the printer (defining the z axis position of the printhead) to the indicated values to keep the distance between the printhead and the structure top layer approximatively the same.

| **Tab. S1\|** 200 µm high and 100 µm wide MNs. | **Tab. S2\|** 600 µm high and 100 µm wide MNs. |
| --- | --- |
| \| **Steps** \| **Base diameter** \| **# of layers** \| **Substrate thickness** \| \| --- \| --- \| --- \| --- \| \| 0 \| 2.5 × 1.1mm \| 5 \| 1900 µm \| \| 1 \| 100 µm \| 10 \| 1910 µm \| \| 2 \| 95 µm \| 10 \| 1928 µm \| \| 3 \| 90 µm \| 10 \| 1946 µm \| \| 4 \| 85 µm \| 10 \| 1964 µm \| \| 5 \| 80 µm \| 10 \| 1982 µm \| \| 6 \| 75 µm \| 10 \| 2000 µm \| \| 7 \| 70 µm \| 10 \| 2018 µm \| \| 8 \| 65 µm \| 10 \| 2036 µm \| \| 9 \| 60 µm \| 10 \| 2054 µm \| \| 10 \| 55 µm \| 10 \| 2072 µm \| \| 11 \| 50 µm \| 10 \| 2090 µm \| \| 12 \| 45 µm \| 10 \| 2108 µm \| \| 13 \| 40 µm \| 10 \| 2126 µm \| \| 14 \| 35 µm \| 10 \| 2144 µm \| \| 15 \| 30 µm \| 10 \| 2162 µm \| | \| **Steps** \| **Base diameter** \| **# of layers** \| **Substrate thickness** \| \| --- \| --- \| --- \| --- \| \| 0 \| 2.5 × 1.1mm \| 5 \| 1900 µm \| \| 1 \| 100 µm \| 30 \| 1910 µm \| \| 2 \| 95 µm \| 30 \| 1960 µm \| \| 3 \| 90 µm \| 30 \| 2010 µm \| \| 4 \| 85 µm \| 30 \| 2060 µm \| \| 5 \| 80 µm \| 30 \| 2110 µm \| \| 6 \| 75 µm \| 30 \| 2160 µm \| \| 7 \| 70 µm \| 30 \| 2210 µm \| \| 8 \| 65 µm \| 30 \| 2260 µm \| \| 9 \| 60 µm \| 30 \| 2310 µm \| \| 10 \| 55 µm \| 30 \| 2360 µm \| \| 11 \| 50 µm \| 30 \| 2410 µm \| \| 12 \| 45 µm \| 30 \| 2460 µm \| \| 13 \| 40 µm \| 30 \| 2510 µm \| \| 14 \| 35 µm \| 30 \| 2560 µm \| \| 15 \| 30 µm \| 30 \| 2610 µm \| |
|  |  |
| **Tab. S3\|** 1000 µm high and 100 µm wide MNs. | **Tab. S4\|** 200 µm high and 300 µm wide MNs. |
| \| **Steps** \| **Base diameter** \| **# of layers** \| **Substrate thickness** \| \| --- \| --- \| --- \| --- \| \| 0 \| 2.5 x 1.1mm \| 5 \| 1900 µm \| \| 1 \| 100 µm \| 50 \| 1910 µm \| \| 2 \| 95 µm \| 50 \| 1993 µm \| \| 3 \| 90 µm \| 50 \| 2076 µm \| \| 4 \| 85 µm \| 50 \| 2159 µm \| \| 5 \| 80 µm \| 50 \| 2242 µm \| \| 6 \| 75 µm \| 50 \| 2325 µm \| \| 7 \| 70 µm \| 50 \| 2408 µm \| \| 8 \| 65 µm \| 50 \| 2491 µm \| \| 9 \| 60 µm \| 50 \| 2574 µm \| \| 10 \| 55 µm \| 50 \| 2657 µm \| \| 11 \| 50 µm \| 50 \| 2740 µm \| \| 12 \| 45 µm \| 50 \| 2823 µm \| \| 13 \| 40 µm \| 50 \| 2906 µm \| \| 14 \| 35 µm \| 50 \| 2989 µm \| \| 15 \| 30 µm \| 50 \| 3072 µm \| | \| **Steps** \| **Base diameter** \| **# of layers** \| **Substrate thickness** \| \| --- \| --- \| --- \| --- \| \| 0 \| 2.7 × 1.1mm \| 5 \| 1900 µm \| \| 1 \| 300 µm \| 6 \| 1910 µm \| \| 2 \| 280 µm \| 6 \| 1922 µm \| \| 3 \| 260 µm \| 6 \| 1934 µm \| \| 4 \| 240 µm \| 6 \| 1946 µm \| \| 5 \| 220 µm \| 6 \| 1958 µm \| \| 6 \| 200 µm \| 6 \| 1970 µm \| \| 7 \| 180 µm \| 6 \| 1982 µm \| \| 8 \| 160 µm \| 6 \| 1994 µm \| \| 9 \| 140 µm \| 6 \| 2006 µm \| \| 10 \| 120 µm \| 6 \| 2018 µm \| \| 11 \| 100 µm \| 6 \| 2030 µm \| \| 12 \| 90 µm \| 6 \| 2042 µm \| \| 13 \| 80 µm \| 6 \| 2054 µm \| \| 14 \| 70 µm \| 6 \| 2066 µm \| \| 15 \| 65 µm \| 6 \| 2078 µm \| \| 16 \| 60 µm \| 6 \| 2090 µm \| \| 17 \| 55 µm \| 6 \| 2102 µm \| \| 18 \| 50 µm \| 6 \| 2114 µm \| \| 19 \| 45 µm \| 6 \| 2126 µm \| \| 20 \| 40 µm \| 6 \| 2138 µm \| \| 21 \| 35 µm \| 6 \| 2150 µm \| \| 22 \| 30 µm \| 6 \| 2162 µm \| |
|  |  |
| **Tab. S5\|** 600 µm high and 300 µm wide MNs. | **Tab. S6\|** 1000 µm high and 300 µm wide MNs. |
| \| **Steps** \| **Base diameter** \| **# of layers** \| **Substrate thickness** \| \| --- \| --- \| --- \| --- \| \| 0 \| 2.7 × 1.1mm \| 5 \| 1900 µm \| \| 1 \| 300 µm \| 18 \| 1910 µm \| \| 2 \| 280 µm \| 18 \| 1942 µm \| \| 3 \| 260 µm \| 18 \| 1974 µm \| \| 4 \| 240 µm \| 18 \| 2006 µm \| \| 5 \| 220 µm \| 18 \| 2038 µm \| \| 6 \| 200 µm \| 18 \| 2070 µm \| \| 7 \| 180 µm \| 18 \| 2102 µm \| \| 8 \| 160 µm \| 18 \| 2134 µm \| \| 9 \| 140 µm \| 18 \| 2166 µm \| \| 10 \| 120 µm \| 18 \| 2198 µm \| \| 11 \| 100 µm \| 18 \| 2230 µm \| \| 12 \| 90 µm \| 18 \| 2262 µm \| \| 13 \| 80 µm \| 18 \| 2294 µm \| \| 14 \| 70 µm \| 18 \| 2326 µm \| \| 15 \| 65 µm \| 18 \| 2358 µm \| \| 16 \| 60 µm \| 18 \| 2390 µm \| \| 17 \| 55 µm \| 18 \| 2422 µm \| \| 18 \| 50 µm \| 18 \| 2454 µm \| \| 19 \| 45 µm \| 18 \| 2486 µm \| \| 20 \| 40 µm \| 18 \| 2518 µm \| \| 21 \| 35 µm \| 18 \| 2550 µm \| \| 22 \| 30 µm \| 18 \| 2582 µm \| | \| **Steps** \| **Base diameter** \| **# of layers** \| **Substrate thickness** \| \| --- \| --- \| --- \| --- \| \| 0 \| 2.7 × 1.1mm \| 5 \| 1900 µm \| \| 1 \| 300 µm \| 30 \| 1910 µm \| \| 2 \| 280 µm \| 30 \| 1960 µm \| \| 3 \| 260 µm \| 30 \| 2010 µm \| \| 4 \| 240 µm \| 30 \| 2060 µm \| \| 5 \| 220 µm \| 30 \| 2110 µm \| \| 6 \| 200 µm \| 30 \| 2160 µm \| \| 7 \| 180 µm \| 30 \| 2210 µm \| \| 8 \| 160 µm \| 30 \| 2260 µm \| \| 9 \| 140 µm \| 30 \| 2310 µm \| \| 10 \| 120 µm \| 30 \| 2360 µm \| \| 11 \| 100 µm \| 30 \| 2410 µm \| \| 12 \| 90 µm \| 30 \| 2460 µm \| \| 13 \| 80 µm \| 30 \| 2510 µm \| \| 14 \| 70 µm \| 30 \| 2560 µm \| \| 15 \| 65 µm \| 30 \| 2610 µm \| \| 16 \| 60 µm \| 30 \| 2660 µm \| \| 17 \| 55 µm \| 30 \| 2710 µm \| \| 18 \| 50 µm \| 30 \| 2760 µm \| \| 19 \| 45 µm \| 30 \| 2810 µm \| \| 20 \| 40 µm \| 30 \| 2860 µm \| \| 21 \| 35 µm \| 30 \| 2910 µm \| \| 22 \| 30 µm \| 30 \| 2960 µm \| |
|  |  |
| **Tab. S7\|** 200 µm high and 500 µm wide MNs. | **Tab. S8\|** 600 µm high and 500 µm wide MNs. |
| \| **Steps** \| **Base diameter** \| **# of layers** \| **Substrate thickness** \| \| --- \| --- \| --- \| --- \| \| 0 \| 3.0 × 1.2mm \| 5 \| 1900 µm \| \| 1 \| 500 µm \| 4 \| 1910 µm \| \| 2 \| 480 µm \| 4 \| 1915 µm \| \| 3 \| 460 µm \| 4 \| 1920 µm \| \| 4 \| 440 µm \| 4 \| 1925 µm \| \| 5 \| 420 µm \| 4 \| 1930 µm \| \| 6 \| 400 µm \| 4 \| 1935 µm \| \| 7 \| 380 µm \| 4 \| 1940 µm \| \| 8 \| 360 µm \| 4 \| 1945 µm \| \| 9 \| 340 µm \| 4 \| 1950 µm \| \| 10 \| 320 µm \| 4 \| 1955 µm \| \| 11 \| 300 µm \| 4 \| 1960 µm \| \| 12 \| 280 µm \| 4 \| 1965 µm \| \| 13 \| 260 µm \| 4 \| 1970 µm \| \| 14 \| 240 µm \| 4 \| 1975 µm \| \| 15 \| 220 µm \| 4 \| 1980 µm \| \| 16 \| 200 µm \| 4 \| 1985 µm \| \| 17 \| 180 µm \| 4 \| 1990 µm \| \| 18 \| 160 µm \| 4 \| 1995 µm \| \| 19 \| 140 µm \| 4 \| 2000 µm \| \| 20 \| 120 µm \| 4 \| 2005 µm \| \| 21 \| 100 µm \| 4 \| 2010 µm \| \| 22 \| 90 µm \| 4 \| 2015 µm \| \| 23 \| 80 µm \| 4 \| 2020 µm \| \| 24 \| 70 µm \| 4 \| 2025 µm \| \| 25 \| 65 µm \| 4 \| 2030 µm \| \| 26 \| 60 µm \| 4 \| 2035 µm \| \| 27 \| 55 µm \| 4 \| 2040 µm \| \| 28 \| 50 µm \| 4 \| 2045 µm \| \| 29 \| 45 µm \| 4 \| 2050 µm \| \| 30 \| 40 µm \| 4 \| 2055 µm \| \| 31 \| 35 µm \| 4 \| 2060 µm \| \| 32 \| 30 µm \| 4 \| 2065 µm \| | \| **Steps** \| **Base diameter** \| **# of layers** \| **Substrate thickness** \| \| --- \| --- \| --- \| --- \| \| 0 \| 3.0 × 1.2mm \| 5 \| 1900 \| \| 1 \| 500 µm \| 24 \| 1910 \| \| 2 \| 480 µm \| 12 \| 1929 \| \| 3 \| 460 µm \| 12 \| 1948 \| \| 4 \| 440 µm \| 12 \| 1967 \| \| 5 \| 420 µm \| 12 \| 1986 \| \| 6 \| 400 µm \| 12 \| 2005 \| \| 7 \| 380 µm \| 12 \| 2024 \| \| 8 \| 360 µm \| 12 \| 2043 \| \| 9 \| 340 µm \| 12 \| 2062 \| \| 10 \| 320 µm \| 12 \| 2081 \| \| 11 \| 300 µm \| 12 \| 2100 \| \| 12 \| 280 µm \| 12 \| 2119 \| \| 13 \| 260 µm \| 12 \| 2138 \| \| 14 \| 240 µm \| 12 \| 2157 \| \| 15 \| 220 µm \| 12 \| 2176 \| \| 16 \| 200 µm \| 12 \| 2195 \| \| 17 \| 180 µm \| 12 \| 2214 \| \| 18 \| 160 µm \| 12 \| 2233 \| \| 19 \| 140 µm \| 12 \| 2252 \| \| 20 \| 120 µm \| 12 \| 2271 \| \| 21 \| 100 µm \| 12 \| 2290 \| \| 22 \| 90 µm \| 12 \| 2309 \| \| 23 \| 80 µm \| 12 \| 2328 \| \| 24 \| 70 µm \| 12 \| 2347 \| \| 25 \| 65 µm \| 12 \| 2366 \| \| 26 \| 60 µm \| 12 \| 2385 \| \| 27 \| 55 µm \| 12 \| 2404 \| \| 28 \| 50 µm \| 12 \| 2423 \| \| 29 \| 45 µm \| 12 \| 2442 \| \| 30 \| 40 µm \| 12 \| 2461 \| \| 31 \| 35 µm \| 12 \| 2480 \| \| 32 \| 30 µm \| 12 \| 2499 \| |
|  |  |
| **Tab. S9\|** 1000 µm high and 500 µm wide MNs. |  |
| \| **Steps** \| **Base diameter** \| **# of layers** \| **Substrate thickness** \| \| --- \| --- \| --- \| --- \| \| 0 \| 3.0 × 1.2mm \| 5 \| 1900 µm \| \| 1 \| 500 µm \| 20 \| 1910 \| \| 2 \| 480 µm \| 20 \| 1945 \| \| 3 \| 460 µm \| 20 \| 1980 \| \| 4 \| 440 µm \| 20 \| 2015 \| \| 5 \| 420 µm \| 20 \| 2050 \| \| 6 \| 400 µm \| 20 \| 2085 \| \| 7 \| 380 µm \| 20 \| 2120 \| \| 8 \| 360 µm \| 20 \| 2155 \| \| 9 \| 340 µm \| 20 \| 2190 \| \| 10 \| 320 µm \| 20 \| 2225 \| \| 11 \| 300 µm \| 20 \| 2260 \| \| 12 \| 280 µm \| 20 \| 2295 \| \| 13 \| 260 µm \| 20 \| 2330 \| \| 14 \| 240 µm \| 20 \| 2365 \| \| 15 \| 220 µm \| 20 \| 2400 \| \| 16 \| 200 µm \| 20 \| 2435 \| \| 17 \| 180 µm \| 20 \| 2470 \| \| 18 \| 160 µm \| 20 \| 2505 \| \| 19 \| 140 µm \| 20 \| 2540 \| \| 20 \| 120 µm \| 20 \| 2570 \| \| 21 \| 100 µm \| 20 \| 2610 \| \| 22 \| 90 µm \| 20 \| 2645 \| \| 23 \| 80 µm \| 20 \| 2680 \| \| 24 \| 70 µm \| 20 \| 2715 \| \| 25 \| 65 µm \| 20 \| 2750 \| \| 26 \| 60 µm \| 20 \| 2785 \| \| 27 \| 55 µm \| 20 \| 2820 \| \| 28 \| 50 µm \| 20 \| 2855 \| \| 29 \| 45 µm \| 20 \| 2890 \| \| 30 \| 40 µm \| 20 \| 2925 \| \| 31 \| 35 µm \| 20 \| 2960 \| \| 32 \| 30 µm \| 20 \| 2995 \| |  |

**
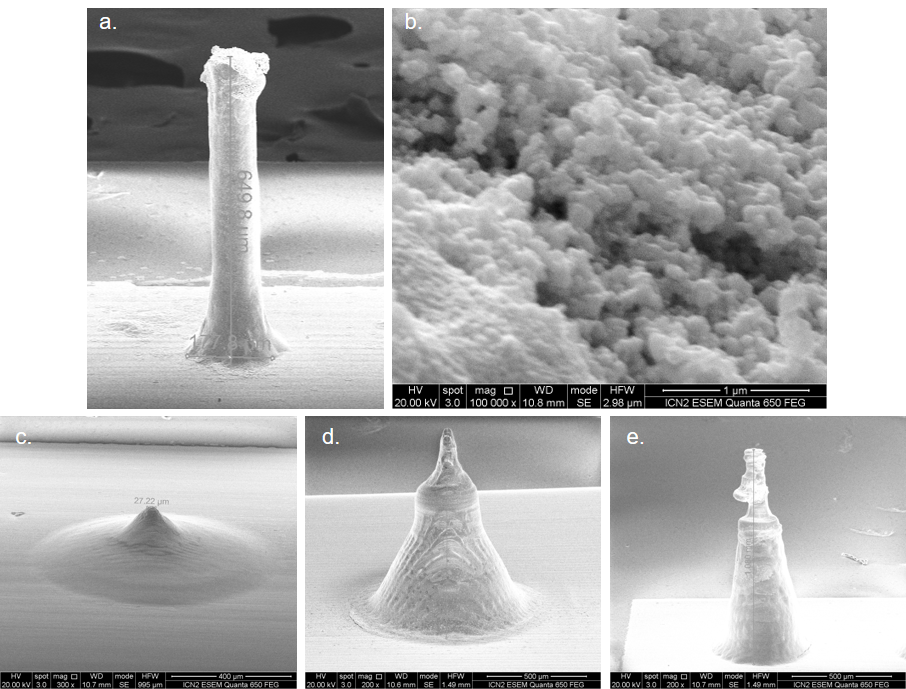
**

**Fig. S1| SEM pictures of MNs structures obtained with the AgNP inkjet printing approach with the heater. a,** example of a vertical structure. **b,** Magnification of the nanoparticles agglomeration inside a crack in the microneedles. **c, d, and e,** examples of microneedles of different dimensions and of defects due to changes in the jetting performance of the printer or to vibrations.

**
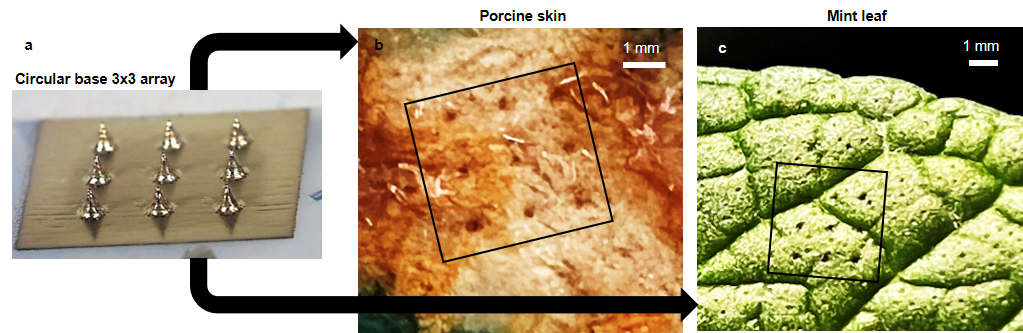
**

**Fig S2| Pictures of the MNs penetration marks left on porcine skin and mint leaves**
